# Swimming Phase-Based Performance Evaluation Using a Single IMU in Main Swimming Techniques

**DOI:** 10.3389/fbioe.2021.793302

**Published:** 2021-12-07

**Authors:** Mahdi Hamidi Rad, Kamiar Aminian, Vincent Gremeaux, Fabien Massé, Farzin Dadashi

**Affiliations:** ^1^ Laboratory of Movement Analysis and Measurement, EPFL, Lausanne, Switzerland; ^2^ Institute of Sport Sciences, University of Lausanne, Lausanne, Switzerland; ^3^ Swiss Olympic Medical Center, Sport Medicine Unit, Division of Physical Medicine and Rehabilitation, Lausanne University Hospital, Lausanne, Switzerland; ^4^ Gait Up S.A., Lausanne, Switzerland; ^5^ Huma Therapeutics Ltd., London, United Kingdom

**Keywords:** sports biomechanics, wearable sensor, swimming, performance evaluation, variable selection

## Abstract

Comprehensive monitoring of performance is essential for swimmers and swimming coaches to optimize the training. Regardless of the swimming technique, the swimmer passes various swimming phases from wall to wall, including a dive into the water or wall push-off, then glide and strokes preparation and finally, swimming up to the turn. The coach focuses on improving the performance of the swimmer in each of these phases. The purpose of this study was to assess the potential of using a sacrum-worn inertial measurement unit (IMU) for performance evaluation in each swimming phase (wall push-off, glide, stroke preparation and swimming) of elite swimmers in four main swimming techniques (i.e. front crawl, breaststroke, butterfly and backstroke). Nineteen swimmers were asked to wear a sacrum IMU and swim four one-way 25 m trials in each technique, attached to a tethered speedometer and filmed by cameras in the whole lap as reference systems. Based on the literature, several goal metrics were extracted from the instantaneous velocity (e.g. average velocity per stroke cycle) and displacement (e.g. time to reach 15 m from the wall) data from a tethered speedometer for the swimming phases, each one representing the goodness of swimmer’s performance. Following a novel approach, that starts from swimming bout detection and continues until detecting the swimming phases, the IMU kinematic variables in each swimming phase were extracted. The highly associated variables with the corresponding goal metrics were detected by LASSO (least absolute shrinkage and selection operator) variable selection and used for estimating the goal metrics with a linear regression model. The selected kinematic variables were relevant to the motion characteristics of each phase (e.g. selection of propulsion-related variables in wall push-off phase), providing more interpretability to the model. The estimation reached a determination coefficient (R^2^) value more than 0.75 and a relative RMSE less than 10% for most goal metrics in all swimming techniques. The results show that a single sacrum IMU can provide a wide range of performance-related swimming kinematic variables, useful for performance evaluation in four main swimming techniques.

## Introduction

Swimming coaches seek comprehensive monitoring of performance to develop and refine a competition model for their top athletes. During a competition, the swimmer goes through several swimming phases from wall to wall, including a dive into the water or wall push-off, then glide and strokes preparation and finally swimming up to the turn at the end of the lap and repeating the same sequence in the next lap. Therefore, to have a comprehensive performance evaluation, studies have focused on various swimming phases, since the swimmers aim to master all of them (Mooney et al., 2016). As the principal goal of a swimmer is to reduce the swimming time by increasing the velocity, performance evaluation goal metrics in different phases are based on time records and velocity. Flight distance (Ruschel et al., 2007), time to 15 m (Vantorre et al., 2010), average velocity per stroke (Dadashi et al., 2015), swimming phase average velocity (Mason and Cossor, 2000), turn time (5 m before to 10 m after the wall) (Mooney et al., 2016) or lap time are examples of common goal metrics.

Recently, wearable IMUs (inertial measurement unit) have been used more for swimming motion analysis in all competitive swimming techniques (Guignard et al., 2017b), because of the challenges of video-based systems application in aquatic environments (Callaway et al., 2010). They are used in a multitude of studies for variable extraction in various swimming phases, such as start (Vantorre et al., 2014), swimming (Davey et al., 2008), and turn (Slawson et al., 2012). Novel orientation analysis algorithms made it possible to estimate the 3-dimensional orientation of IMU with high accuracy by fusing accelerometer, gyroscope and magnetometer data (Madgwick et al., 2011). This approach is implemented in swimming for inter-segmental coordination assessment (Guignard et al., 2017a), posture recognition (Wang et al., 2019) and intra-stroke velocity (Worsey et al., 2018). In another study, a new analysis approach is proposed and trunk elevation, body balance, and body rotation are used as new indices for swimming analysis (Félix et al., 2019; Morouço et al., 2020). Considering the significance of phase related kinematic variables, we have recently proposed a macro-micro approach for swimming analysis using IMUs (Hamidi Rad et al., 2021). In our approach, swimming bouts, laps and swimming technique are detected in macro analysis. Afterwards in micro level, each lap is segmented into swimming phases of wall push-off (*Push*), glide (*Glid*), strokes preparation (*StPr*), swimming (*Swim*) and turn (*Turn*) from wall to wall. In the next level of micro analysis, the kinematic variables within each swimming phase (micro variables) are extracted from IMU data.

These studies show there is still a substantial undiscovered potential for kinematic variable extraction with IMUs in swimming analysis. However, the association between the swimming kinematic variables extracted by IMU and the above-mentioned goal metrics is still unclear. Furthermore, as the variables provided by the IMU are claimed to be associated with the swimmers’ performance, they can be used for estimating the goal metrics of performance evaluation. As a result, the relationship between IMU kinematic variables and goal metrics is yet to be studied to prove IMU potential not only for swimming kinematic variable extraction, but also for performance evaluation and training optimization.

The main objective of this study is to find the association between swimming kinematics extracted using a sacrum-worn IMU and goal metrics in different swimming phases. We hypothesized that the micro variables extracted from IMU data are associated with the goal metrics used for performance evaluation, regardless of the swimming technique. Following the macro-micro approach for swimming analysis (Hamidi Rad et al., 2021), within each swimming phase (*Push*, *Glid*, *StPr* and *Swim*), we selected the kinematic variables that are highly associated with goal metrics. We then used the selected kinematics to estimate the goal metrics. Using the underlying model we can explains how kinematics determine the performance.

## Materials and Methods

### Measurement Setup and Protocol

**FIGURE 1:**
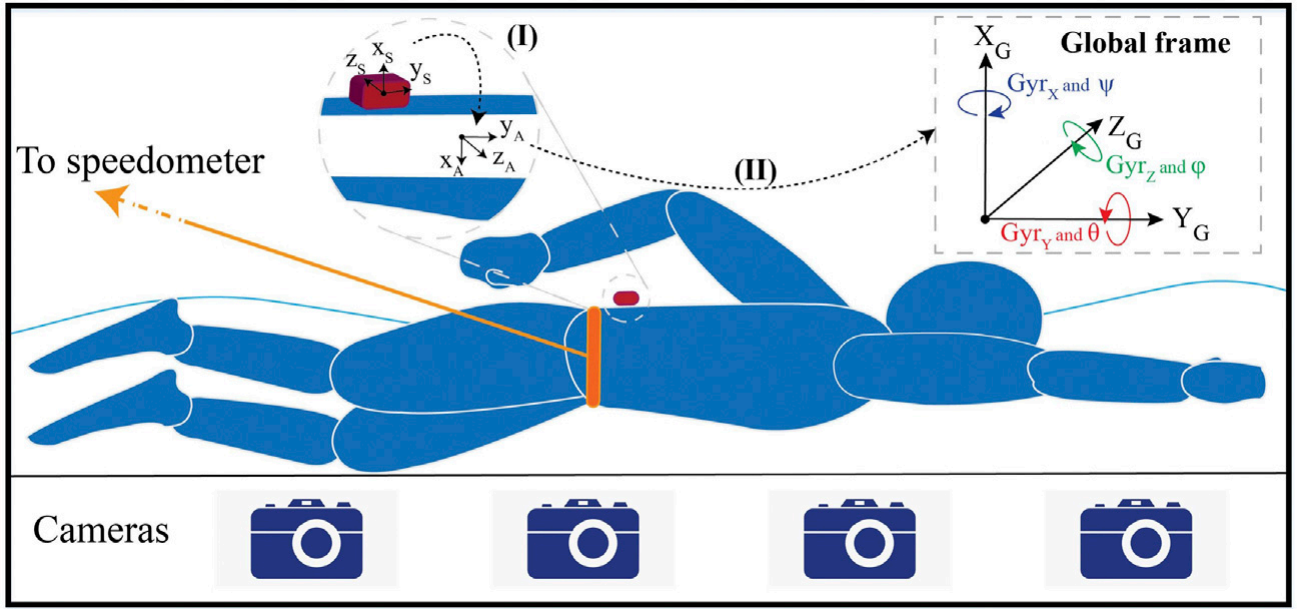
Measurement setup including one IMU attached to the sacrum, four cameras to capture the whole lap and tethered speedometer to record swimmer’s displacement and velocity. IMU data is transferred from sensor frame (x,y,z)_S_, first to anatomical frame (x,y,z)_A_ using functional calibration (I), and then to the global frame (X,Y,Z)_G_ using the gradient-descend based optimization algorithm (II). The global axes of acceleration, angular velocity and angles are displayed in the figure.

Nineteen elite swimmers took part in this study, whose attributes are shown in Table 1. They were informed of the procedure and gave their written consent prior to participation. This study was approved by the EPFL human research ethics committee (HREC, No: 050/2018). One IMU (Physilog^®^ IV, GaitUp, CH.) was attached to swimmer’s sacrum, using waterproof band (Tegaderm, 3M Co., USA). The sensor contained a 3D gyroscope (±2000 °/s) and 3D accelerometer (±16 g), with a sampling rate of 500 Hz (Figure 1). A functional calibration was performed after sensor installation with simple movements in land (upright standing and squats) before the measurement to make the data independent of sensor placement on swimmer’s body (Dadashi et al., 2013). During the measurements, the swimmers were asked to perform four one-way trials in each swimming technique (i.e. front crawl, breaststroke, butterfly, backstroke) with a progressive velocity (70–100%) in a 25 m indoor pool, starting with wall push-off inside water. The trials were separated with 1-min rests, and the total duration of the measurement was around 1 hour per swimmer.

**TABLE 1:** Statistics of the study participants. All variables are presented as mean ± standard deviation. Record50m is the average and standard deviation of 50 m record of the swimmers separately for each swimming technique.

Male	Female	Age (yrs)	Height (cm)	Weight (kg)	Record50m (s)	
9	10	19.5 ± 2.7	177.5 ± 7.5	67.9 ± 8.3	Front crawl	25.85 ± 1.65
					Breaststroke	34.76 ± 3.87
					Butterfly	28.55 ± 2.47
					Backstroke	30.19 ± 1.88

Two systems were used as references in our study to validate the goal metrics estimated by the IMU. The first one was a set of four 2-D cameras (GoPro Hero 7 Black, GoPro Inc., US) used for detecting the swimming phases. The cameras synchronized with the IMU, using the LED light of a push-button (Hamidi Rad et al., 2021) were attached to the pool wall (distributed along the length of the pool) to videotape all the lap from wall to wall underwater with a 60 Hz rate (Figure 1). The second reference system was a tethered speedometer (SpeedRT^®^, ApLab, Rome, Italy), attached with a belt to the waist of the swimmer. The speedometer calculated the displacement and velocity of the swimmer at a rate of 100 Hz and was used for finding the reference values of goal metrics in different swimming phases. As the speedometer was installed on the starting block above the swimmer’s level, it caused a parallax problem (Le Sage et al., 2011). Since the device level difference with respect to the still pool water was known (62 ± 1 cm), the velocity projection along the swimming direction was separated as the forward velocity of the swimmer.

### Performance Evaluation

**FIGURE 2:**
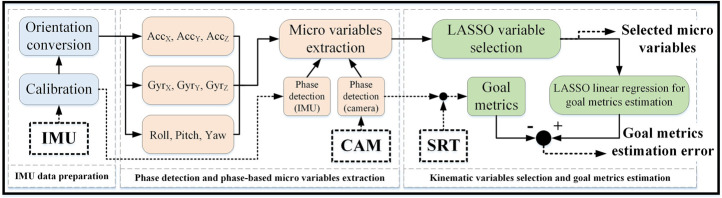
Flowchart of the performance evaluation algorithm. IMU data preparation including IMU calibration and expressing data in the global frame **(left),** phase detection by cameras (CAM) or IMU calibrated data and micro variable extraction from IMU data in global frame **(middle)** and variable selection from micro variables and the goal metrics estimation **(right).** The actual goal metrics are defined and extracted from the velocity and displacement data by tethered speedometer (SRT) during swimming phases separated by the cameras (CAM).

The general flowchart for performance evaluation is outlined in Figure 2. The algorithm includes three parts: 1) IMU data preparation 2) phase detection and phase-based micro variables extraction, 3) kinematic variable selection and goal metrics estimation. IMU data preparation aims to transfer the data to the global frame to achieve the true motion data of swimmer’s sacrum. Then we divided each lap into four phases of *Push*, *Glid*, *StPr* and *Swim* by camera or IMU (Hamidi Rad et al., 2021). In order to observe the error induced by IMU-based phase detection, the rest of the analysis was done once with swimming phases detected by cameras and once by the IMU for comparison, the results of which are illustrated in supplementary materials. Using the data in global frame (acceleration ( $Acc_{X}$ , $Acc_{Y}$ , $Acc_{Z}$ ), angular velocity ( $Gyr_{X}$ , $Gyr_{Y}$ , $Gyr_{Z}$ ) and orientation (Roll, Pitch, Yaw)) within the detected phases, we extracted the micro variables of each phase.

In the third part of this approach, we used the extracted phase-based micro variables to estimate the goal metrics. First, LASSO (least absolute shrinkage and selection operator) variable selection is used to rank and select the micro variables with higher importance (Fonti and Belitser, 2017). Using the speedometer and camera data, several goal metrics are extracted on the velocity and displacement of the swimmer in different swimming phases. These goal metrics are representatives of how well the swimmer performed in the corresponding phase. Finally, we used the selected micro variables to estimate the goal metrics. The principal outputs of this analysis are the selected variables and the error of using them for goal metrics estimation.

#### IMU Data Preparation

First, the data was calibrated for offset, scale and non-orthogonality (Ferraris et al., 1995). As explained in *Measurement Setup and Protocol*, a functional calibration is also performed before each measurement trial. The goal of this calibration is to transform the data from sensor frame ${\left(x,y,z\right)}_{S}$ to anatomical frame ${\left(x,y,z\right)}_{A}$ (Figure 1-I). Following that, the data is ready to be expressed in the global frame. The swimmers were asked to hold an upright posture in water before lap start for 5 seconds to find the initial orientation of the sacrum with respect to the pool. The changes from the initial orientation are estimated by angular velocity integration from gyroscope data and corrected with acceleration using a gradient-descend based optimization algorithm (Madgwick et al., 2011). The algorithm provides the orientation changes in quaternion *q* [represented by four elements ( $q_{1},\;q_{2},\;q_{3},\;q_{4}$ )] and use them to convert the accelerometer and gyroscope data from anatomical frame [ ${\left(x,y,z\right)}_{A}$ ] to global frame [ ${\left(X,Y,Z\right)}_{G}$ ] (Figure 1-II), expressed in Eqs 1, 2.

$$Acc_{G}=q⊗\left[0\;Acc_{A}\right]⊗q^{T}$$ (1)

$$Gyr_{G}=q⊗\left[0\;Gyr_{A}\right]⊗q^{T}$$ (2)

Where $Acc_{A}$ and $Acc_{G}$ are the acceleration in anatomical and global frame respectively, ⊗ represents quaternion multiplication and $q^{T}$ is the transpose of the quaternion *q*. The same notation holds true for gyroscope data in Eq. 2. Moreover, by changing quaternions into Euler angles, roll ( θ ), pitch ( φ ) and yaw ( ψ ) angles could be found (Eq. 3). The angles θ , φ and ψ are defined respectively around the longitudinal, mediolateral, and anterior-posterior axes of swimmer’s sacrum.

$$\{\begin{array}{c} \psi \;=A\tan2\left(2q_{2}q_{3}-2q_{1}q_{4},2q_{1}^{2}+2q_{2}^{2}-1\right)\;\\ \theta \;=\;-\sin^{-1}\left(2q_{2}q_{4}+2q_{1}q_{3}\right)\\ \varphi \;=\;A\tan2\left(2q_{3}q_{4}-2q_{1}q_{2},2q_{1}^{2}+2q_{4}^{2}-1\right)\; \end{array}$$ (3)

#### Phase-Based Micro Variables

For IMU-based detection of swimming phases, we used a macro-micro approach in our previous study, started from swimming bouts detection down to lap segmentation into swimming phases (Hamidi Rad et al., 2021). Using the acceleration, angular velocity and orientation data in global frame, various kinematic variables based on motion biomechanics in every swimming phase are defined. As frequently discussed in the literature, fast swimming depends on 1) the ability to generate high propulsive forces, 2) the ability to keep the correct posture for reducing the drag, while 3) swimming with the highest efficiency (Toussaint and Truijens, 2005). Therefore, knowledge of the propulsion, posture and efficiency is relevant to optimize swimming performance. We related the extracted micro variables to one of these three categories (Table 2). We also added a fourth group for the variables related to the durations and rates of motion, which did not fit into the previous three categories. For example stroke rate in *Swim* phase which is not necessarily a sign of high or low propulsion, good or bad posture and high or low efficiency but it is widely used for performance evaluation (Siirtola et al., 2011; Beanland et al., 2014).

**TABLE 2:** Categories and description of the phase-based micro variable defined on IMU data in global frame. The name of the functions used for micro variables extraction are abbreviated in parentheses.

Category	Description	Micro variables
Propulsion	Variables related to the amount of propulsion generated by the swimmer	Mean (*Mean*), range (*Range*) and standard deviation (*SD*) of $Acc_{X}$ , $Acc_{Y}$ and $Acc_{Z}$ . Maximum (*Max*), integral (*Int*), and momentum change (*Momentum*) of $Acc_{Y}$
Posture	Variables related to the body posture and drag effects on swimmer’ body	*Mean*, *Range* and *SD* of θ and φ
Efficiency	Variables related to the efficiency of motion which can reflect in acceleration	Ratio of positive $Acc_{Y}$ to |Acc| (*Eff_dir*) or to negative $Acc_{Y}$ (*Eff*), distance per stroke (*DPS*) in *Swim* phase
Duration/rate	Variables related to the duration of a phase or the rate of movement	*Mean*, *Range* and *SD* of $Gyr_{X}$ , $Gyr_{Y}$ and $Gyr_{Z}.$ phases and cycles duration. Kick rate and count in *StPr* phase. Stroke rate and count in *Swim* phase

We extracted the micro variables by extremum detection, integration or calculation of the average, range and standard deviation of the signal. The variables defined per stroke in *Swim* phase need a cycle separation algorithm. For front crawl and backstroke, the duration between the two successive positive peaks on the longitudinal angular velocity in anatomical frame ( $Gyr_{y}$ ) is one cycle (Dadashi et al., 2013). The same method is used with mediolateral angular velocity in anatomical frame ( $Gyr_{z}$ ) for cycle separation of breaststroke and butterfly techniques.

#### Goal Metrics

**FIGURE 3:**
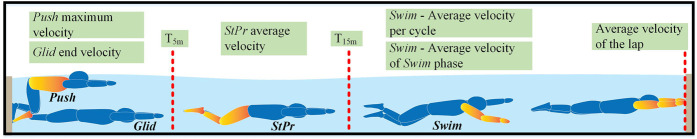
The defined goal metrics for different swimming phases from wall to wall.

We extracted eight goal metrics from the tethered speedometer data i.e. the velocity and displacement of the swimmer, from wall to wall within the swimming phases detected on the cameras (Figure 3).

1. *Push* maximum velocity: the highest velocity during the lap is generated at start, as the swimmer can reach a velocity much greater than other swimming phases (Shimadzu et al., 2008). During *Push* phase, the maximum velocity reached is used to assess wall push-off (Stamm et al., 2013). We use this value as the goal metric for *Push* phase.
2. *Glid* end velocity: the velocity decreases during *Glid* phase because of water drag. The swimmer should keep the streamlined horizontal posture and start *StPr* phase at the right time before losing too much velocity (Vantorre et al., 2014). So we considered the velocity of the swimmer at the end of *Glid* phase as the goal metric for this phase.
3. *StPr* average velocity: the average velocity of the swimmer during *StPr* lower limbs actions is shown to have a negative correlation with 15-m time of the swimmer (Cossor and Mason, 2001). We used it as the goal metric for *StPr* phase.

During *Swim* phase, the performance of the swimmer can be studied per cycle or in the whole phase. Thus two goal metrics are defined in this phase:

4. *Swim*—average velocity per cycle: the average velocity of the swimmer per cycle provides valuable information of swimmer’s performance in every cycle (Dadashi et al., 2015).
5. *Swim*—average velocity of *Swim* phase: for looking at all the cycles together, the average velocity of the whole *Swim* phase is used as the second goal metric for this phase.

We also used three more goal metrics based on the literature, which include more than one phase.

6. $T_{5m}$ : normally *Glid* phase finishes before reaching 5 m from the wall when the swimmer starts by wall push-off in all swimming techniques. The time it takes the swimmer to reach 5 m from the wall is a goal metric (Zatsiorsky et al., 1979), which shows the combination of swimmer’s performance during *Push* and *Glid* phases.
7. $T_{15m}$ : 15 m is the limit for the swimmer to re-surface (except for breaststroke technique) according to FINA (Federation International de Natation) rules. So the time it takes to reach 15 m from the wall is a goal metric referring to underwater phases (*Push*, *Glid* and *StPr*) (Vantorre et al., 2010).
8. Lap average velocity: considering all the phases together, average velocity of the lap (determined by lap time) is the final goal metric, displaying the overall performance of the swimmer in all phases (Davey et al., 2008; Mooney et al., 2016).

Among the defined goal metrics, *Push* maximum velocity is calculated with a peak detection algorithm in *Push* phase. The rest of the goal metrics only rely on the beginning or end of swimming phases, which are already obtained by phase detection.

#### Association Between Micro Variables and Goal Metrics

After extracting the micro variables from IMU and goal metrics from speedometer and camera data, we look for association between every goal metric with the micro variables of its corresponding phase or phases. For example, *Push* maximum velocity is associated with *Push* phase micro variables. For goal metrics involving more than one phase, such as $T_{5m}$ , $T_{15m}$ and lap average velocity related to *Push/Glid*, *Push/Glid/StPr* and all phases respectively, the micro variables from the relevant phases were used.

To identify the variables with higher significance, we ran a variable selection algorithm. In the first step, we normalized each variable and removed the multicollinearity between them using variance inflation factors (VIF) (Mansfield and Helms, 1982). LASSO variable selection is then applied over the variables related to each goal metric, to select the ones of higher importance. LASSO is a forward-looking variable selectin method for regression, which improves both the estimation accuracy and the interpretability of the model (Muthukrishnan and Rohini, 2017). It ranks the variables and allocates a wight to each one based on their significance in the regression model. Among the selected variables, we neglected the ones with a relative weight less than 5% because of their less important role. Moreover, to quantify the contribution of each category to the regression model, we summed the relative weights of variables from each category (propulsion, posture, efficiency and duration/rate).

Once the significant variables were identified, we utilized them to estimate the goal metrics by a LASSO regression model with leave-one-out cross-validation to avoid overfitting (Berrar, 2018). The cross validated determination coefficient ( $R^{2}$ ) is reported as a metric of association between true values (reference values from speedometer) and the estimated value (output of the models). The error between the true and estimated values of goal metrics is analyzed using the root mean square of error (*RMSE*) and its relative value in percent.

## Results

A sample size analysis based on a previous study (Dadashi et al., 2012) that used the same speedometer and measurement protocol for velocity estimation is performed. Considering a power of 80% (β = 0.2) and 95% (α = 0.05) confidence interval, we reached a sample size of 64 for this study. Since the models are generated using the data from all swimmers pooled together, the number of observations used to estimate all goal metrics, except for average velocity of the cycle in *Swim* phase was 76 samples. The overall number of cycles used for estimating the average velocity per cycle in *Swim* phase was 1,166, 627, 695 and 1,052 for front crawl, breaststroke, butterfly and backstroke respectively.

### Goal Metrics Estimation

The cross-validated values (R^2^, RMSE and the relative RMSE in percent) of LASSO regression model used for estimating the corresponding goal metric are reported in Table 3 for each goal metric. Table 3 shows that LASSO regression model fits the data with an R^2^ value more than 0.75 for most goal metrics in all swimming techniques. The RMSE of the regression are less than 0.15 ms (11%) for all goal metrics defined over velocity and less than 0.21 s (7%) and 0.52 s (5%) for $T_{5m}$ and $T_{15m}$ respectively. The highest value of relative RMSE belongs to *Glid* end velocity with 11.1%, while the relative error is less than 10% in all other cases. The results are also calculated with swimming phases found by cameras for comparison in supplementary materials (Supplementary Table SA1).

**TABLE 3:** The results of evaluating LASSO regression for goal metrics estimation. The determination coefficient (R^2^) and root mean square of error (RMSE) and the relative RMSE (in %) of regression are reported for each swimming technique.

Goal metric	Front crawl		Breaststroke	
	R^2^	RMSE (%)	R^2^	RMSE (%)
*Push* maximum velocity (m/s)	0.74	0.140 (5.7)	0.75	0.131 (5.3)
*Glid* end velocity (m/s)	0.76	0.123 (10.1)	0.64	0.139 (11.1)
*StPr* average velocity (m/s)	0.72	0.075 (4.4)	0.58	0.058 (5.9)
*Swim—*average velocity per cycle (m/s)	0.89	0.050 (8.3)	0.84	0.044 (5.7)
Average velocity of *Swim* phase (m/s)	0.90	0.044 (2.7)	0.71	0.061 (5.3)
$T_{5m}$ (s)	0.64	0.158 (7.6)	0.74	0.209 (6.9)
$T_{15m}$ (s)	0.75	0.369 (4.3)	0.81	0.430 (6.7)
Lap average velocity (m/s)	0.95	0.032 (2.4)	0.85	0.038 (3.4)
	**Butterfly**		**Backstroke**	
*Push* maximum velocity (m/s)	0.71	0.149 (5.9)	0.72	0.107 (4.9)
*Glid* end velocity (m/s)	0.80	0.111 (9.1)	0.84	0.104 (6.4)
*StPr* average velocity (m/s)	0.75	0.152 (6.7)	0.75	0.079 (5.3)
*Swim—*average velocity per cycle (m/s)	0.88	0.067 (4.9)	0.89	0.076 (5.7)
Average velocity of *Swim* phase (m/s)	0.79	0.049 (3.3)	0.73	0.056 (4.3)
$T_{5m}$ (s)	0.63	0.209 (7.0)	0.71	0.202 (6.4)
$T_{15m}$ (s)	0.79	0.344 (4.6)	0.77	0.521 (5.0)
Lap average velocity (m/s)	0.86	0.049 (3.3)	0.80	0.063 (4.6)

### Micro-Variables Selection

The selected variables for each goal metric estimation during front crawl technique are listed in Table 4. Same tables for other swimming techniques are brought in supplementary materials (Supplementary Tables A2–A4). Among acceleration axes, $Acc_{Y}$ and its related variables [e.g. *Mean* ( $Acc_{Y}$ ), *Max* ( $Acc_{Y}$ ), *Int* ( $Acc_{Y}$ )] are more selected for different goal metrics. $Gyr_{Z}$ and φ related variables seem to be more associated with the defined goal metrics than other axes of orientation in front crawl technique. For $T_{5m}$ _,_ $T_{15m}$ and lap average velocity, a mixture of variables from corresponding phases are selected, some of which were already selected for the specific goal metric of these phases.

**TABLE 4:** The selected variables for estimating each goal metric for front crawl technique, written in the order of relative weights. The variables are written in the order of their relative weights. For the abbreviated name of functions, see Table 2.

Goal metric	Selected variables
*Push* maximum velocity	*Range* ( φ ), *SD* ( φ ), *Int* ( $Acc_{Y}$ ), *Momentum* ( $Acc_{Y}$ ), *Range* ( $Acc_{Y}$ ), *Max* ( $Acc_{Y}$ ), *Mean* ( $Gyr_{Z}$ ), *Eff* ( $Acc_{Y}$ )
*Glid* end velocity	*Glid* duration, *Momentum* ( $Acc_{Y}$ ), *Int* ( $Acc_{Y}$ ), *Range* ( $Acc_{Y}$ ), *Range* ( φ ), *Mean* ( φ )
*StPr* average velocity	*Mean* ( $Acc_{Y}$ ), *Eff* ( $Acc_{Y}$ ), *Eff_dir* ( $Acc_{Y}$ ), *SD* ( $Acc_{Y}$ ) , number of kicks, *StPr* duration
*Swim—*average velocity per cycle	Cycle duration, DPS, *Mean* ( φ ) per cycle
Average velocity of *Swim* phase	Stroke rate, Mean ( φ ), number of strokes, *SD* ( $Acc_{Y}$ ), *Range* ( θ )
$T_{5m}$	*Momentum* ( $Acc_{Y}$ ) in *Glid*, *Max* ( $Gyr_{Z}$ ) in *Push*, *SD* ( φ ) in *Glid*, *Range* ( φ ) in *Push, Max* ( $Gyr_{Z}$ ) in *Glid*
$T_{15m}$	*Glid* duration, *Range* ( φ ) in *StPr*, *SD* ( $Gyr_{Z}$ ) in *StPr*, *SD* ( $Acc_{Y}$ ) in *Push*, *StPr* kick rate, *Momentum* ( $Acc_{Y}$ ) in *Push*
Lap average velocity	Stroke rate, number of strokes, *Max* ( $Acc_{Y}$ ) in *Push*, *Mean* ( $Acc_{Y}$ ) in *Glid*, *Mean* ( φ ) in *Swim*, number of kicks in *StPr*

**FIGURE 4:**
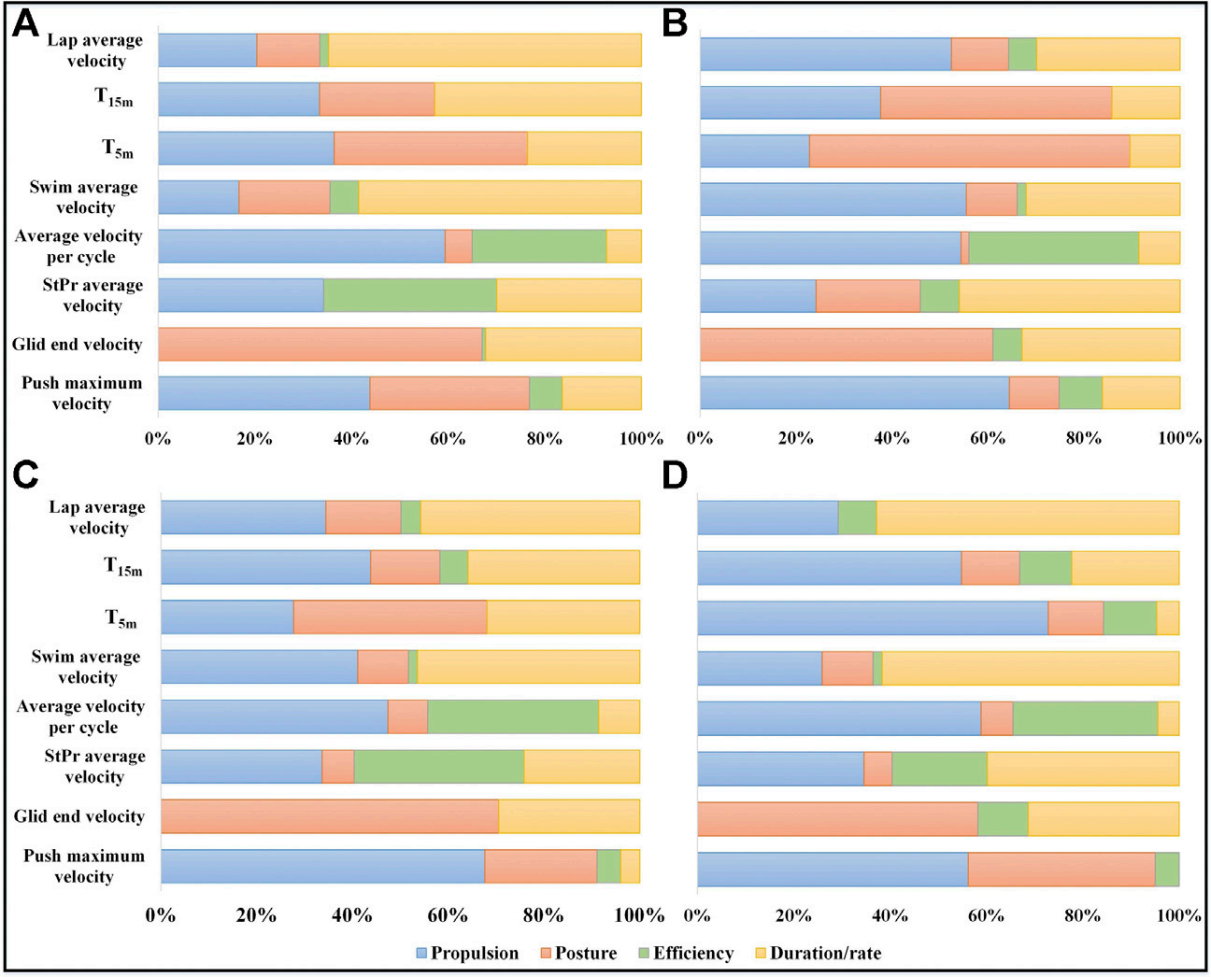
Variable categories contribution to goal metrics estimation for front crawl **(A)**, breaststroke **(B)**, butterfly **(C)** and backstroke **(D)**. The contribution of each category (propulsion: blue, posture: orange, efficiency: green, duration/rate: yellow) is represented in percent for estimating the corresponding goal metric. The results are based on the variables with higher than 5% relative weight in LASSO variable selection.

The overall contribution of each category in estimating the goal metrics is illustrated in Figure 4 for all four swimming techniques. It is observable that propulsion category plays an important role in *Push* phase, while posture-related variables are more selected in *Glid* phase. *StPr* phase is less affected by efficiency compared to other categories. Efficiency and propulsion categories are both significant in determining the average velocity per cycle in *Swim* phase. Duration/rate category is dominant in estimating average velocity of *Swim* phase and lap average velocity. $T_{5m}$ and $T_{15m}$ are affected mainly by a mixture of propulsion, posture and duration/rate categories depending on the swimming technique.

## Discussion

In this research, we studied the association between IMU micro variables and the performance evaluation goal metrics found by camera and speedometer during the swimming phases from wall to wall in four main swimming techniques. The obtained results confirmed our hypothesis that micro variables extracted from a single IMU placed at sacrum within each phase are associated with the corresponding goal metrics used generally for performance evaluation. As a result, using a single IMU would be enough for performance evaluation in main swimming techniques. Micro variables, showing strong association with the goal metrics, were identified thanks to LASSO variable selection and used for predicting the goal metrics.

### Goal Metrics Estimation

The selected kinematic variables within each swimming phase were used for estimating the corresponding goal metrics (Table 3). Estimating the *Push* maximum velocity and *Glid* end velocity showed similar results in different swimming techniques, as the two initial phases are the same for them (only for backstroke, the swimmer has a supine posture). The relative RMSE is the highest for *Glid* end velocity estimation (11%) because this goal metric has the lowest value in the whole lap. In the *StPr* phase, the average velocity shows a high amount of variability among the swimmers, and determination coefficient (i.e. the proportion of the variance of the true goal metric value explained by the regression model) is relatively lower for it (less than 0.8 in all techniques) compared to other goal metrics in all techniques, because a linear model is not efficient enough in reflecting the variation of this goal metric, and a non-linear model might estimate it better.

Average velocity per cycle is estimated in all techniques with a determination coefficient more than 0.84 and an RMSE less than 0.076 m/s and relative error less than 6%. However, estimating the average velocity of the whole *Swim* phase achieved poorer results (R^2^ of 0.71–0.90 in different techniques). As estimating each cycle average velocity is more accurate in all techniques, the average value of all cycles in *Swim* phase can also be used for estimating *Swim* phase average velocity. The regression models for estimating $T_{5m}$ show less accuracy (R^2^ less than 0.80 in different techniques), making it difficult to trust the estimation results. Depending on swimming technique and swimmers’ pace, they might start *StPr* phase earlier than 5 m from the wall. So $T_{5m}\;$ is partly affected by *StPr* phase and using only *Push* and *Glid* phases might not be enough for estimation. On the contrary, the first three phases (*Push*, *Glid* and *StPr*) finish before 15 m from the wall and using them for estimating the $T_{15m}$ results in more accurate regression models (R^2^ more than 0.75 in different techniques). Finally, the lap average velocity is estimated using a selection of the kinematic variables from all phases with a relatively small error (RMSE less than 0.063 m/s for all techniques). The results have been only slightly improved when using cameras for phase detection (section 1 of Supplementary Materials).

### Micro Variables Selection

As shown in Table 4 and Figure 4 during the *Push* phase, the kinematic variables related to φ and $Acc_{Y}$ are ranked as more important, which shows the significance of keeping the right posture and generating high propulsion in *Push* phase. The *Mean* ( $Gyr_{Z}$ ) and *Eff* ( $Acc_{Y}$ ) are selected at last. The weight contribution of *Push* kinematic variables can be categorized more in propulsion and posture groups, which is the same for all techniques (Figures 4A–D), as the *Push* movement is the same. During *Glid* phase, phase duration is chosen the first, since the longer the *Glid* phase is, the more velocity will be lost. *Momentum* ( $Acc_{Y}$ ) and *Int* ( $Acc_{Y}$ ) are also considered important since they represent the effect of water drag on swimmer’s body. High *Range* ( φ ) and *Mean* ( φ ) during *Glid* are a sign of bad posture, which causes more drag. In terms of categories, none of the micro variables can be categorized in propulsion because *Glid* phase does not include any propulsive motion. As a result, the categories of posture and duration/rate are the dominant groups in this phase, regardless of the technique.

*StPr* phase has the highest amount of velocity variation on speedometer data and the average velocity during this phase is related to a combination of forward acceleration, accelerating efficiency, number of kicks and phase duration. Two types of efficiency-related variables are selected for this phase. *Eff* ( $Acc_{Y}$ ) represents the ratio of positive to all forward acceleration and *Eff_dir* ( $Acc_{Y}$ ) is the ratio of forward acceleration to the acceleration norm. Since this phase includes strong kicking, generating the highest amount of acceleration in forward direction ( $Acc_{Y}$ ) with respect to other axes is selected as an important variable. *StPr* phase is the same for front crawl, butterfly and backstroke as it includes butterfly kicks in all of them. Figures 4A,C,D also shows similar categories of propulsion, efficiency and duration/rate for the variables selected in this phase. For breaststroke technique, *StPr* phase includes one upper limbs cycle followed by a lower limb action and the posture related variables are also important compared to other categories (Figure 4B).

For *Swim* phase goal metrics, the average velocity per stroke is mainly associated with the duration of each cycle and the DPS. The *Mean* ( φ ) is also selected which relates to the swimmer’s posture. This selection is the same in all swimming techniques (Figures 4B–D) as the average velocity per stroke can be estimated by dividing the DPS by the cycle duration. The second goal metric of *Swim* phase is the average velocity of the whole phase. The variables related to the rate and number of strokes are more dominant as the swimmers increase the stroke rate for fast swimming. The *SD* ( $Acc_{Y}$ ), *Mean* ( φ ) and *Range* ( θ ) are other kinematic variables selected for estimating this goal metric, highlighting the significance of consistent propulsion and body posture in *Swim* phase. As a result, the three categories of duration/rate, posture and propulsion are more pronounced for estimating *Swim* phase average velocity in all techniques.

$T_{5m}$ , $T_{15m}$ and lap average velocity are dependent on more than one phase, and the variable selection algorithm picks a number of variables from each phase. Most of the selected variables for these goal metrics were already selected for relevant phases such as selecting *Momentum* ( $Acc_{Y}$ ) of *Glid* for $T_{5m}$ , *Glid* duration for $T_{15m}$ or stroke rate for lap average velocity, proving the significance of such variables even in a larger scale. Moreover, this shows the dependence of overall swimmer’s performance on their local performance in each phase. Among the techniques, $T_{5m}$ and $T_{15m}$ are estimated with a mixture of propulsion, posture and duration/rate categories in front crawl, breaststroke and butterfly, whereas during backstroke, the propulsion is dominant for both goal metrics. This emphasises on the tendency of the swimmers to longer underwater phases in backstroke (De Jesus et al., 2011), that asks for highly propulsive butterfly kicks.

With an overall observation on Figure 4, it is noted that the dominant categories in swimming phases are in line with the swimming phase biomechanics. *Push* phase asks for high propulsion, and *Glid* phase is more about keeping the right posture to avoid the drag. *StPr* phase is a combination of propulsion, posture and efficiency. Since the variable selection algorithm chooses the best variables for goal metric estimation, the variables which have the strongest relationship with the goal metrics are selected. As a result, we cannot assert that the rest of the variables are of no importance in swimming. For example, the *DPS* and cycle duration were dominant in estimating the average velocity per cycle in *Swim* phase, while no one can ignore the importance of orientation-related variables (e.g. θ angle) (Psycharakis and Sanders, 2010) or propulsion (Toussaint, 2002) in this phase. However, having a longer *DPS* in a shorter cycle duration is the result of correct orientation and high propulsion so the selected variables include other variable categories implicitly.

This study shows that a single sacrum IMU can provide kinematic variables relevant to the performance of the swimmer, in different techniques and phases for performance evaluation without using complex instrumentation such as speedometers or cameras. This offers new tools for training, where for example output of the IMU can be transferred to a mobile application for coaches and swimmers to easily follow the progress of the swimmers. Although using wearables induces more drag on swimmer body (Magalhaes et al., 2015), it needs extremely less effort than cameras for preparation and use, and it overcomes many of the limits of video-based systems (Callaway et al., 2010). The kinematic variables that were found dominant in our study were already analyzed using IMU of video-based methods but their relationship with the goal metrics were not studied. Swimmer’s posture during *Push* and *Glid* (Pereira et al., 2015), *Glid* duration (Guimaraes and Hay, 1985), *StPr* kicking rate (Shimojo et al., 2014), *Swim* stroke rate (Beanland et al., 2014) or DPS (Bächlin et al., 2008) are examples of the micro variables that were found relevant to performance, and we also found them significant in this study.

Both male and female swimmers were included for generating the results of this study to have a larger, more variant dataset. Comparing the swimmers due to their individual differences is out of the scope of our study. The estimations are done over all swimming velocities so the results are valid for 70–100 percent of swimmers’ paces. The synchronization error between the three systems of IMU, cameras and speedometer is a source of error in this study. Since tethered speedometer was used as reference in this study, the measurements were done over one-way trials without turn and turn motion is not evaluated. In this study, we used linear regression to have interpretable models highlighting the main variables correlated to the goal metrics. More complex non-linear models could be used if the goal is more accurate prediction of goal metrics.

## Conclusion

Using the IMU data, we extracted numerous kinematic variables related to propulsion, posture, efficiency and duration/rate of motion in four main swimming phases, associated with the goal metrics defined over velocity and time of swimming in each swimming phase. These kinematic variables were biomechanically interpretable and were able to predict the goal metrics using LASSO linear regression. The generated models fit the data with an R^2^ value more than 0.75 for most goal metrics. The RMSE of the regression were less than 0.15 ms and 11% for goal metrics defined over velocity and 0.52 s and 7.6% for goal metrics defined over time. Our study shows that a single sacrum-worn IMU has the potential to evaluate the swimmer performance in different swimming phases in line with standard goal metrics. Practically, our proposed method can be useful for coaches to identify the weakness and strength of their swimmers and track their progress during training sessions with a single IMU. This study can be continued with implementation of the regression models on new dataset for validation, using more complex models (e.g. non-linear regression) for better goal metric estimation, completing the analysis for diving start and turn and using other sensor locations for estimation accuracy comparison.

## Data Availability

The raw data supporting the conclusion of this article will be made available to qualified researcher, without undue reservation.
