# Supplementary material for: Swimming Phase-Based Performance Evaluation Using a Single IMU in Main Swimming Techniques
Source: Front Bioeng Biotechnol. 2021 Dec 7;9:793302. doi: 10.3389/fbioe.2021.793302 (PMC8688996; doi:10.3389/fbioe.2021.793302)
Supplement: Supplementary file 1 [file DataSheet1.PDF]

Supplementary Material

1 Performance evaluation (phase detection via IMU vs. camera)

We evaluate the impact of phase detection using IMU on goal metrics estimation using LASSO regression as the estimator. This can be achieved by incorporating IMU-based swimming phases and camera-based swimming phase (CAM) separately and incorporating them as input to the regression model. The cross-validated values (R^2, RMSE and the relative RMSE in percent) of regression are reported for each swimming technique using both methods for comparison in table A1.

In the worst case (*Push* maximum velocity of butterfly technique), R^2 has decreased 0.08, while it is affected less than 0.05 for most of other goal metrics. Moreover, the relative RMSE has increased no more than 3.5% for any goal metric. This shows that the error of IMU phase detection is small enough, not to affect the goal metrics estimation accuracy.

Table A1 – The results of evaluating LASSO regression for goal metrics estimation using IMU-based or camera-based (CAM) phase detection. The determination coefficient (R^2) and root mean square of error (RMSE) and the relative RMSE (in %) of regression are reported for each swimming technique.

Goal metric	Front crawl				Breaststroke			
	CAM		IMU		CAM		IMU	
	R^2	RMSE **(%)**	R^2	RMSE **(%)**	R^2	RMSE **(%)**	R^2	RMSE **(%)**
Push maximum velocity **(m/s)**	0.80	0.133 **(5.4)**	0.74	0.140 **(5.7)**	0.81	0.114 **(4.6)**	0.75	0.131 **(5.3)**
Glid end velocity **(m/s)**	0.83	0.105 **(8.7)**	0.76	0.123 **(10.1)**	0.70	0.135 **(11.0)**	0.64	0.139 **(11.1)**
StPr average velocity **(m/s)**	0.72	0.075 **(4.4)**	0.72	0.075 **(4.4)**	0.64	0.054 **(5.8)**	0.58	0.058 **(5.9)**
Swim – average velocity per cycle **(m/s)**	0.96	0.029 **(4.8)**	0.89	0.050 **(8.3)**	0.86	0.062 **(5.9)**	0.84	0.044 **(5.7)**
Average velocity of *Swim* phase **(m/s)**	0.90	0.044 **(2.7)**	0.90	0.044 **(2.7)**	0.76	0.059 **(5.1)**	0.71	0.061 **(5.3)**
T_{5m} **(s)**	0.67	0.155 **(7.5)**	0.64	0.158 **(7.6)**	0.77	0.206 **(6.8)**	0.74	0.209 **(6.9)**
T_{15m} **(s)**	0.80	0.345 **(4.0)**	0.75	0.369 **(4.3)**	0.82	0.430 **(6.7)**	0.81	0.430 **(6.7)**
Lap average velocity **(m/s)**	0.95	0.031 **(2.3)**	0.95	0.032 **(2.4)**	0.90	0.042 **(3.8)**	0.85	0.038 **(3.4)**
	**Butterfly**				**Backstroke**			
Push maximum velocity **(m/s)**	0.79	0.110 **(3.8)**	0.71	0.149 **(5.9)**	0.75	0.105 **(4.8)**	0.72	0.107 **(4.9)**
Glid end velocity **(m/s)**	0.81	0.104 **(8.5)**	0.80	0.111 **(9.1)**	0.86	0.104 **(6.5)**	0.84	0.104 **(6.4)**

StPr average velocity **(m/s)**	0.75	0.153 **(6.7)**	0.75	0.152 **(6.7)**	0.77	0.070 **(5.2)**	0.75	0.079 **(5.3)**
Swim – average velocity per cycle **(m/s)**	0.88	0.067 **(4.9)**	0.88	0.067 **(4.9)**	0.89	0.076 **(5.7)**	0.89	0.076 **(5.7)**
Average velocity of *Swim* phase **(m/s)**	0.79	0.048 **(3.3)**	0.79	0.049 **(3.3)**	0.73	0.056 **(4.3)**	0.73	0.056 **(4.3)**
T_{5m} **(s)**	0.66	0.204 **(6.8)**	0.63	0.209 **(7.0)**	0.72	0.197 **(6.2)**	0.71	0.202 **(6.4)**
T_{15m} **(s)**	0.86	0.342 **(3.5)**	0.79	0.344 **(4.6)**	0.82	0.499 **(4.8)**	0.77	0.521 **(5.0)**
Lap average velocity **(m/s)**	0.86	0.048 **(3.3)**	0.86	0.049 **(3.3)**	0.84	0.052 **(3.8)**	0.80	0.063 **(4.6)**

2 Parameter selection

Table A2 to A4 represent the parameters that were selected using LASSO method for each swimming phase in breaststroke, butterfly and backstroke techniques. Refer to section 2.1 in the main text for definition of anatomical axes.

Table A2 – Table of the selected parameters for each goal metric in breaststroke technique. The parameters are ordered according to their weights in the regression model.

Goal metric	Selected parameters
Push maximum velocity	*Int* (Acc_Y), *Max* (Gyr_Z), *Max* (Acc_Y), *Range* (Acc_Y), *SD* (φ), *Momentum* (Acc_Y), *Eff* (Acc_Y) , *SD* (Acc_Y), *Mean* (Acc_Y)
Glid end velocity	*Glid* duration, *Int* (Acc_Y), *Momentum* (Acc_Y), *Max* (Acc_Y), *Range* (φ), *Mean* (φ), *Range* (Acc_Y), *SD* (φ)
StPr average velocity	*Range* (Acc_Y), *StPr* duration, *Range* (φ), *Mean* (φ), *Eff* (Acc_Y), *SD* (Gyr_Y), *Mean* (Gyr_Y),
Swim – average velocity per cycle	Cycle duration, DPS, *SD* (Acc_Y), *Range* (Gyr_Z), *Range* (Acc_Y), *Range* (Gyr_Y)
Average velocity of *Swim* phase	*SD* (Acc_Y), Stroke rate, number of strokes, *Range* (φ), *SD* (φ)
T_{5m}	*Max* (Gyr_Z) in *Push*, *Momentum* (Acc_Y) in *Glid*, *Max* (Gyr_Z) in *Glid*, *SD* (φ) in *Glid*, *Range* (φ) in *Push*
T_{15m}	*SD* (Acc_Y) in *StPr*, *Range* (Acc_Y) in *Glid*, *Mean* (Gyr_Z) in *Glid*, *Range* (φ) in *Push*, *SD* (φ) in *Push*, *Range* (Acc_Y) in *StPr*, *Max* (Gyr_Z) in *Push*, *Max* (Acc_Y) in *Glid*
Lap average velocity	Stroke rate, *Max* (Acc_Y) in *StPr*, *SD* (Acc_Y) in *Swim*, number of strokes, *Max* (Acc_Y) in *Push*, *SD* (θ) in *Swim*, *Momentum* (Acc_Y) in *Push*, *Eff* (Acc_Y), *Max* (Acc_Y) in *Glid*

Table A3 – Table of the selected parameters for each goal metric in butterfly technique. The parameters are ordered according to their weights in the regression model.

Goal metric	Selected parameters
Push maximum velocity	*SD* (φ), *Int* (Acc_Y), *SD* (Acc_Y), *Momentum* (Acc_Y), *Max* (Gyr_Z), *Mean* (Acc_Y), *Max* (Acc_Y), *Eff* (Acc_Y)
Glid end velocity	*Glid* duration, *Int* (Acc_Y), *Range* (φ), *Range* (Gyr_Z), *Mean* (φ), *Max* (Acc_Y),
StPr average velocity	*Range* (Acc_Y), *Eff_dir*(Acc_Y), *Mean* (Gyr_Y), *Eff* (Acc_Y), *Range* (Gyr_Y)
Swim – average velocity per cycle	Cycle duration, DPS, *SD* (Acc_Y), *Range* (φ), *SD* (Gyr_Z)
Average velocity of *Swim* phase	*SD* (Acc_Y), Stroke rate, number of strokes, *Range* (φ), *SD* (φ)
T_{5m}	*Range* (Gyr_Z) in *Glid*, *Range* (Acc_Y) in *Glid*, *Range* (φ) in *Push*, *Max* (Gyr_Z) in *Push*, *Momentum* (Acc_Y) in *Push*, *Push* duration, *SD* (Acc_Y) in *Push*, *Mean* (φ) in *Glid*
T_{15m}	*Glid* duration, *Range* (Acc_Y) in *StPr*, *SD* (Acc_Y) in *StPr*, *SD* (Acc_Y) in *Push*, *Mean* (φ) in *Push*, *Momentum* (Acc_Y) in *Glid*, *SD* (Gyr_Z) in *StPr*, *Mean* (Acc_Y) in *StPr*
Lap average velocity	Stroke rate, *SD* (Acc_Y) in *Swim*, number of strokes, *Max* (Acc_Y) in *Push*, *SD* (Gyr_Z) in *StPr*, *Range* (φ) in *Swim*, *SD* (φ) in *Swim*

Table A4 – Table of the selected parameters for each goal metric in backstroke technique. The parameters are ordered according to their weights in the regression model.

Goal metric	Selected parameters
Push maximum velocity	*Max* (Acc_Y), *Mean* (φ), *SD* (φ), *Momentum* (Acc_Y), *SD* (Acc_Y), *Max* (Gyr_Z), *Mean* (Acc_Y), *Int* (Acc_Y)
Glid end velocity	*Int* (Acc_Y), *Momentum* (Acc_Y), *Mean* (Gyr_Z), *Glid* duration, *SD* (φ), *SD* (Acc_Y), *Range* (φ)
StPr average velocity	*Range* (Acc_Y), *Eff*(Acc_Y), *SD* (Gyr_Z), *StPr* duration, *Range* (Gyr_Y)
Swim – average velocity per cycle	Cycle duration, DPS, *SD* (Acc_Y)
Average velocity of *Swim* phase	Stroke rate, *SD* (Acc_Y), number of strokes, *Range* (φ)
T_{5m}	*Momentum* (Acc_Y) in *Push*, *SD* (Acc_Y) in *Push*, *Push* duration, *Mean* (φ) in *Glid*, *Mean* (φ) in *Push*
T_{15m}	*Max* (Acc_Y) in *Push*, *Momentum* (Acc_Y) in *Push*, *SD* (Acc_Y) in *StPr*, *Eff*(Acc_Y) in *StPr*, *Glid* duration, *Int* (Acc_Y) in *Glid*, *SD* (Acc_Y) in *Glid*, *Range* (Gyr_Z) in *StPr*,
Lap average velocity	Stroke rate, *Momentum* (Acc_Y) in *Push*, *Max* (Acc_Y) in *Push*, *Range* (Gyr_Z) in *StPr*

3 Glossary of terms

Here is the table of glossary of all the terms used for swimming performance evaluation using one sacrum IMU.

Table A5 – Table of glossary

**Term**	**Definition**
IMU	Inertial measurement unit
CAM	Camera
Acc	Acceleration data (m/s^2)
Gyr	Angular velocity data ($°/s$)
Acc_X, Acc_Y, Acc_Z	Acceleration on X, Y or Z axis of global frame
Gyr_X, Gyr_Y, Gyr_Z	Angular velocity on X, Y or Z axis of global frame
φ	Pitch angle
θ	Roll angle
ψ	Yaw angle
q	Quaternion
LASSO	least absolute shrinkage and selection operator
RMSE	Root mean square error
Swimming bout	The swimming parts (in any swimming technique) during a training session that includes one or more laps.
Swimming lap	The wall-to-wall period of swimming (in any swimming technique) that starts with wall push-off and ends when the swimmer touches the wall.
Swimming technique	The technique of swimming which is one among this list: Front crawl, Breaststroke, Butterfly, Backstroke
Swimming phase	Each lap is divided in four swimming phases (wall push-off, glide, strokes preparation and swimming)
Push	Wall push-off phase
Glid	Glide phase
StPr	Strokes preparation phase
Swim	Swimming phase
T_{5m}	Time to reach 5 meters from the wall
T_{15m}	Time to reach 15 meters from the wall
SD	The standard deviation of a parameter
Max	Maximum value of a parameter
Int	The integral of a parameter
DPS	Distance per stroke
Eff	Efficiency defined as the ratio of positive to negative forward acceleration
Eff_dir	Directional efficiency defined as the ratio of forward acceleration to total acceleration
